# *Polygonum multiflorum* Thunb. Hot Water Extract Reverses High-Fat Diet-Induced Lipid Metabolism of White and Brown Adipose Tissues in Obese Mice

**DOI:** 10.3390/plants10081509

**Published:** 2021-07-23

**Authors:** Ra-Yeong Choi, Mi-Kyung Lee

**Affiliations:** 1 Department of Agricultural Biology, National Institute of Agricultural Sciences, Rural Development Administration, Wanju 55365, Korea; fkdud1304@korea.kr; 2 Department of Food and Nutrition, Sunchon National University, Suncheon 57922, Korea

**Keywords:** anti-obesity, Heshouwu, *Polygonum multiflorum* Thunb., white adipose tissue, brown adipose tissue

## Abstract

The purpose of the present study was to determine whether an anti-obesity effect of a *Polygonum multiflorum* Thunb. hot water extract (PW) was involved in the lipid metabolism of white adipose tissue (WAT) and brown adipose tissue (BAT) in high-fat diet (HFD)-induced C57BL/6N obese mice. Mice freely received a normal diet (NCD) or an HFD for 12 weeks; HFD-fed mice were orally given PW (100 or 300 mg/kg) or garcinia cambogia (GC, 200 mg/kg) once a day. After 12 weeks, PW (300 mg/kg) or GC significantly alleviated adiposity by reducing body weight, WAT weights, and food efficiency ratio. PW (300 mg/kg) improved hyperinsulinemia and enhanced insulin sensitivity. In addition, PW (300 mg/kg) significantly down-regulated expression of carbohydrate-responsive element-binding protein (ChREBP) and diacylglycerol O-acyltransferase 2 (DGAT2) genes in WAT compared with the untreated HFD group. HFD increased BAT gene levels such as adrenoceptor beta 3 (ADRB3), peroxisome proliferator-activated receptor γ (PPARγ), hormone-sensitive lipase (HSL), cluster of differentiation 36 (CD36), fatty acid-binding protein 4 (FABP4), PPARγ coactivator 1-α (PGC-1α), PPARα, and carnitine palmitoyltransferase 1B (CPT1B) compared with the NCD group; however, PW or GC effectively reversed those levels. These findings suggest that the anti-obesity activity of PW was mediated via suppression of lipogenesis in WAT, leading to the normalization of lipid metabolism in BAT.

## 1. Introduction

Obesity is a chronic complex disease caused by a persistent positive energy balance in which energy intake exceeds energy expenditure [1]. In mammals, adipose tissues are classified into three types: white adipose tissue (WAT), brown adipose tissue (BAT), or beige adipose tissue. WAT stores extra energy in a triglyceride (TG) form, while BAT is responsible for dissipation of energy as heat through thermogenesis to maintain body temperature and energy balance [2]. BAT activates thermogenesis in response to cold exposure and overfeeding, thereby contributing to the control of whole-body energy expenditure and body fat contents [3,4]. For this reason, activation of BAT is expected to provide a therapeutic effect on obesity and related metabolic disorders [5].

*Polygonum multiflorum* Thunb. (PM), called Heshouwu in China and East Asia, and Fo-ti in North America, is popular in clinical practice [6]. PM has been used to treat oxidative stress and age-related diseases in traditional medicine [7]. Pharmacological studies revealed that PM attenuates diabetes-induced osteoporosis [8], oxidative stress-induced liver injury [7], focal cerebral ischemia [9], and breast cancer [10], while promoting learning and memory ability [11]. PM possesses treatment effects on hyperlipidemia [12], non-alcoholic fatty liver disease (NAFLD) [13], and atherosclerosis [14]. Our previous study showed the anti-adiposity effects of a PM ethanol extract in 3T3-L1 adipocytes and high-fat diet (HFD)-induced obese mice [15]. In clinical application, PM is usually administrated as a water decoction containing hydrophilic components [16]. However, the possible role of a PM hot water extract (PW) in the lipid metabolic activities of WAT and BAT has never been reported. The present study examined the potential beneficial effects of PW in an HFD-induced obese animal model.

## 2. Results

### 2.1. Effects of PW on Body Weight and Food Intake

The average initial body weight of experimental groups was about 20 g (Table 1). After 7 weeks of HFD feeding, the body weight was significantly increased compared to that of the NCD group. However, the PW300 group showed a remarkable decrease of body weight compared with the HFD group after the 6-week experimental periods. The body weight gain in the HFD group was 16.00 ± 0.61 g over the 12-week study, whereas the PW300 and GC200 groups had markedly reduced total body weight gain, 19.9% and 16.7%, respectively, compared with the HFD group (Table 1). Food intake in the HFD group was significantly lower (12.7%) than that of the NCD group (Table 2). PW and GC did not affect food intake, but the food efficiency ratios (FER) in the NCD, PW300, and GC200 groups were reduced compared with the HFD group (Table 2).

### 2.2. Effects of PW on Relative Adipose Tissue Weight and Adipocyte Size

**Figure 1:**
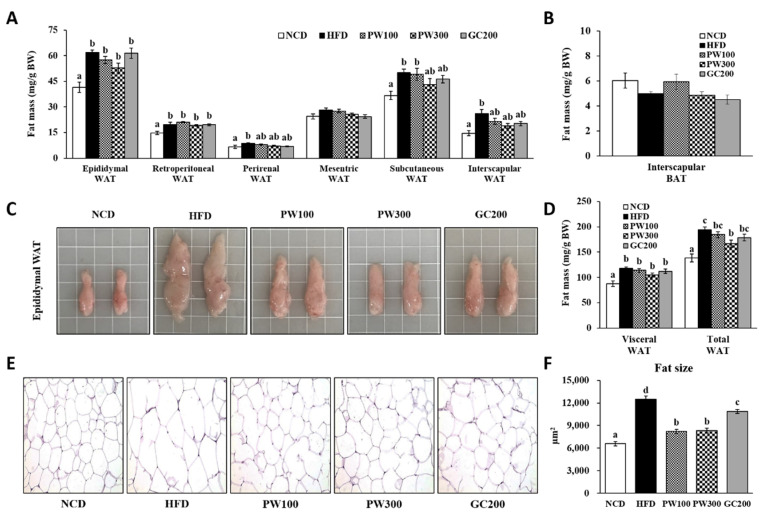
Effects of *Polygonum multiflorum* Thunb. hot water extract on WAT weights (**A**), BAT weight (**B**), representative photographs of epididymal WAT (**C**), visceral and total WAT weights (**D**), H&E microscopic images of epididymal WAT (**E**), fat size of epididymal WAT (**F**) in HFD-induced obese mice. Data are expressed as means ± SE. Values with different superscript letters are significantly different at *p* < 0.05 as determined by one-way ANOVA followed by Tukey’s HSD test. Magnifications × 200. WAT, white adipose tissue; visceral WAT, the sum of epididymal WAT, retroperitoneal WAT, perirenal WAT, and mesenteric WAT; total WAT, the sum of visceral WAT, subcutaneous WAT, and interscapular WAT; BAT, brown adipose tissue.

All relative WAT weights, exclusive of mesenteric WAT, in the HFD group were significantly increased compared with those of the NCD group (Figure 1A,D). Conversely, the PW300 and GC200 groups tended to have lower weights of perirenal, subcutaneous, and interscapular WAT compared with those of the HFD group. Total WAT weights were significantly reduced by PW administration in a dose-dependent manner compared with the HFD group. No statistical significances were detected among the relative interscapular BAT weights of the groups (Figure 1B). The HFD group showed hypertrophy of the epididymal WAT, which was attenuated by treatment with PW and GC (Figure 1C). Moreover, the HFD group showed a larger adipocyte diameter in epididymal WAT, which was recovered following treatment with PW and GC (Figure 1E). Furthermore, the PW100, PW300, and GC200 groups had markedly reduced increases in HFD-induced fat size in the epididymal WAT, by 34.1, 33.2, and 12.8%, respectively (Figure 1F).

### 2.3. Effects of PW on Lipid Metabolism and Inflammatory Gene Expression in Epididymal WAT

**Figure 2:**
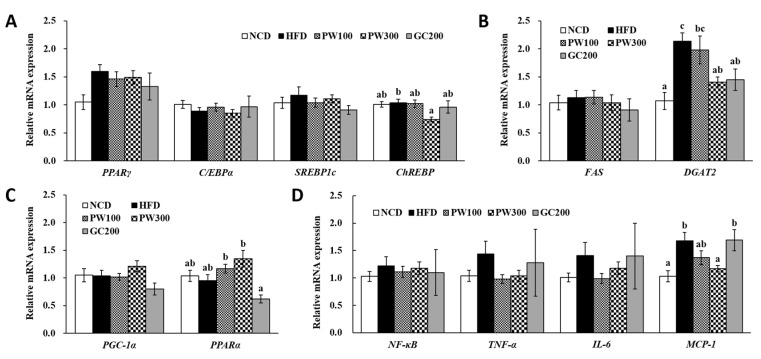
Effects of *Polygonum multiflorum* Thunb. hot water extract on adipogenic and lipogenic transcriptional factor (**A**), lipogenic genes (**B**), FA oxidative transcriptional factor (**C**), and inflammatory gene (**D**) expressions of epididymal WAT in HFD-induced obese mice. Data are expressed as means ± SE. Values with different superscript letters are significantly different at *p* < 0.05 as determined by one-way ANOVA followed by Tukey’s HSD test.

No significant differences were observed in the expressions of peroxisome proliferator-activated receptor γ (*PPARγ*), CCATT/enhancer-binding protein α (*C/EBPα*), sterol regulatory element-binding protein 1c (*SREBP1c*), fatty acid synthase (*FAS*), *PPARγ* coactivator 1-α (*PGC-1α*), nuclear factor-κB (*NF-κB*), tumor necrosis factor-α (*TNF-α*), and interleukin 6 (*IL-6*) genes in epididymal WAT among the groups (Figure 2A–D). The PW300 group had the lowest carbohydrate-responsive element-binding protein (*ChREBP*) mRNA expression, which was significantly down-regulated from that of the HFD group (Figure 2A). The diacylglycerol O-acyltransferase 2 (*DGAT2*) expression in HFD-fed mice was elevated compared with the NCD mice (Figure 2B). The PW300 and GC200 groups had significantly down-regulated *DGAT2* expression, by 31.9% and 30.0%, respectively, compared with that in the HFD group. Moreover, the PW100 and PW300 groups tended towards higher *PPARα* gene expression compared with the HFD group (Figure 2C). Moreover, monocyte chemoattractant protein-1 (*MCP-1*) expression was notably up-regulated, by 63.1%, in the HFD group compared with the NCD group; however, it had a tendency to down-regulate by 18.5% and 30.4%, respectively, in the PW100 and PW300 group compared with the HFD group (Figure 2D).

### 2.4. Effects of PW on Lipid Metabolism Gene Expression in Interscapular BAT

**Figure 3:**
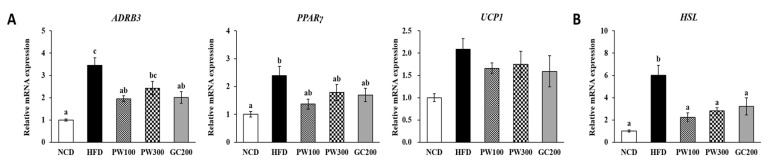
Effects of *Polygonum multiflorum* Thunb. hot water extract on thermogenic (**A**) and lipolytic (**B**) gene expressions in interscapular BAT in HFD-induced obese mice. Data are expressed as means ± SE. Values with different superscript letters are significantly different at *p* < 0.05 as determined by one-way ANOVA followed by Tukey’s HSD test.

**Figure 4:**
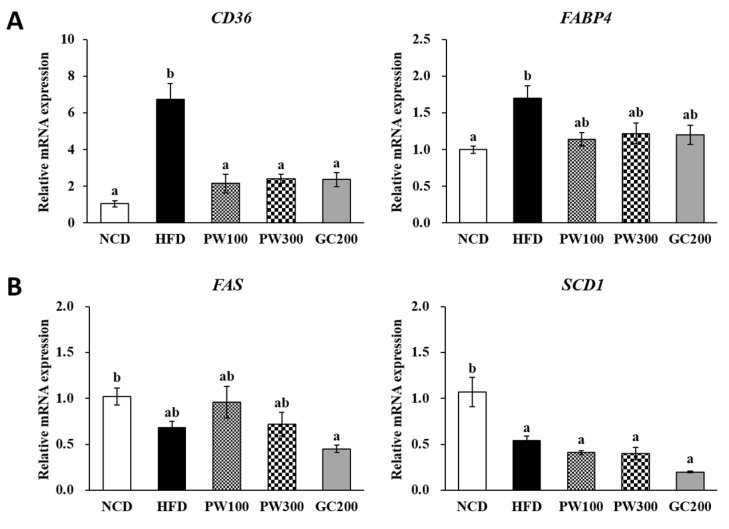
Effects of *Polygonum multiflorum* Thunb. hot water extract on FA uptake (**A**) and FA synthesis (**B**)-related gene expression in interscapular BAT in HFD-induced obese mice. Data are expressed as means ± SE. Values with different superscript letters are significantly different at *p* < 0.05 as determined by one-way ANOVA followed by Tukey’s HSD test.

**Figure 5:**
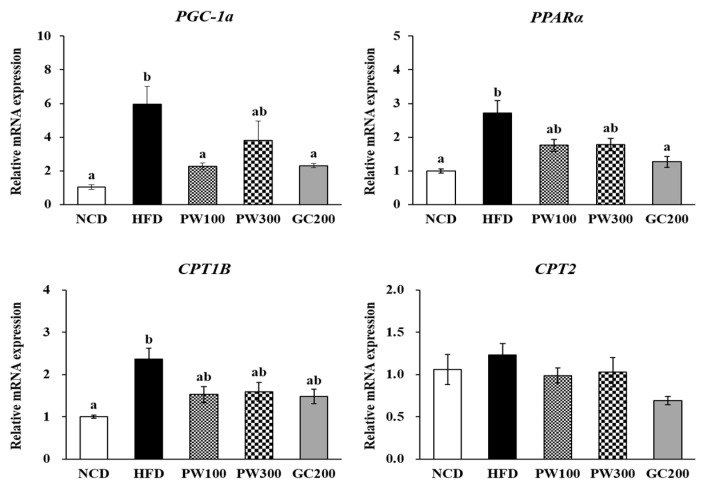
Effects of *Polygonum multiflorum* Thunb. hot water extract on FA oxidation-related gene expression in interscapular BAT in HFD-induced obese mice. Data are expressed as means ± SE. Values with different superscript letters are significantly different at *p* < 0.05 as determined by one-way ANOVA followed by Tukey’s HSD test.

As shown in Figure 3, Figure 4 and Figure 5, the HFD group had elevated gene expression in interscapular BAT genes, such as adrenoceptor beta 3 (*ADRB3*), *PPARγ*, hormone-sensitive lipase (*HSL*), cluster of differentiation 36 (*CD36*), fatty acid-binding protein 4 (*FABP4*), *PGC-1α*, *PPARα*, and carnitine palmitoyltransferase 1B (*CPT1B*), and those elevated levels were recovered to that in the NCD group by treatment with PW (100 and 300 mg/kg) or GC (200 mg/kg). The *FAS* mRNA level was slightly reduced, and the stearoyl-coenzyme A desaturase 1 (*SCD1*) mRNA level was significantly reduced in the HFD group compared to the NCD group. PW and GC did not affect these levels (Figure 4B). Furthermore, no changes were seen in uncoupling protein 1 (*UCP1*) and *CPT2* gene expressions among the groups (Figure 3A and Figure 5).

### 2.5. Effects of PW on Serum Biochemical Parameters and Lipid Profiles

The serum alanine aminotransferase (ALT) level was significantly higher (1.48-fold) in the HFD group than that in the NCD group, whereas the serum ALT level was slightly decreased in the PW100, PW300, and GC200 groups by 6.7%, 28.8%, and 18.6%, respectively, compared to the HFD group (Table 2). No significant differences were observed in serum aspartate aminotransferase (AST) levels among the groups (Table 2). The serum FFA concentration was unaltered by the HFD, but it tended to decrease in the PW300 group compared to the HFD group and was suppressed in the GC200 group (Table 2). The serum HDL-cholesterol (HDL-C) level was 17.6% higher in the HFD group compared with the NCD group (Table 2). No marked differences were detected in the serum TG and total cholesterol (TC) concentrations or the HDL-cholesterol (HDL-C) to TC ratio (HTR) among the groups (Table 2).

### 2.6. Effects of PW on Hyperinsulinemia and Insulin Resistance

**Figure 6:**
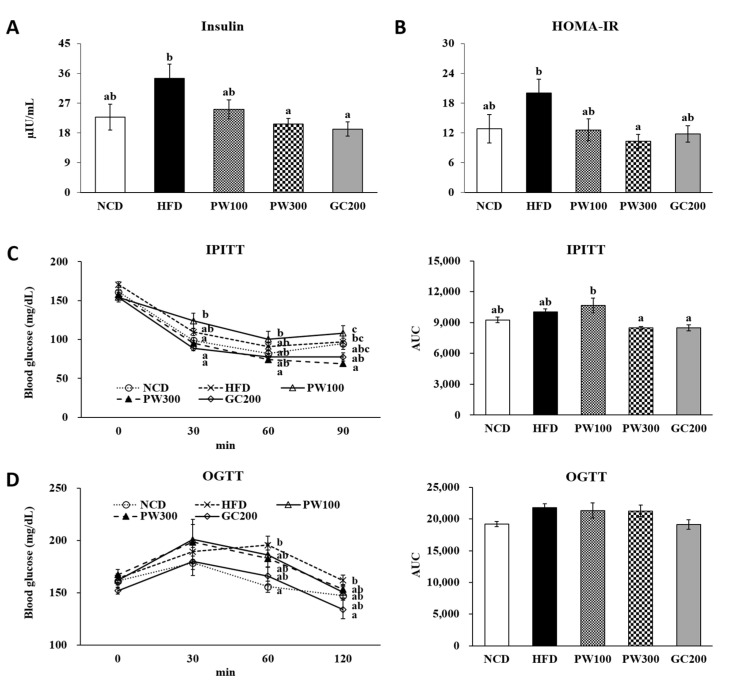
Effect of *Polygonum multiflorum* Thunb. hot water extract on serum insulin level (**A**), HOMA-IR index (**B**), IPITT (**C**), and OGTT (**D**) test in HFD-induced obese mice. Data are expressed as means ± SE. Values with different superscript letters are significantly different at *p* < 0.05 as determined by one-way ANOVA followed by Tukey’s HSD test. Homeostasis model assessment of insulin resistance (HOMA-IR) = fasting serum insulin (µIU/mL) × fasting serum glucose (mmol/L)/22.5. IPITT, intraperitoneal insulin tolerance test; OGTT, oral glucose tolerance test.

Although there were no significant differences among the fasting serum glucose levels of the study groups (Table 2), the fasting serum insulin level and the homeostasis model assessment of insulin resistance (HOMA-IR) index were slightly elevated in the HFD group, approximately 1.5-fold that of the NCD group (Figure 6A,B). In contrast, treatment of the mice with PW (300 mg/kg) resulted in significant reductions in the insulin level and the HOMA-IR index, indicating improvements in hyperinsulinemia and insulin resistance were induced by the HFD.

As shown Figure 6C, there were no changes in the initial blood glucose levels in all groups, but 30 and 90 min following the insulin injection, the HFD group tended to have an increased blood glucose level compared with that of the NCD group. However, the PW300 group exhibited a similar effect to that of the GC200 group and tended to decrease the blood glucose level compared with that of the HFD group. Especially, blood glucose at 90 min showed a significant down-regulation in the PW300 group. Indeed, both the PW300 and GC200 groups had slightly reduced intraperitoneal insulin tolerance test (IPITT) areas under the curve (AUCs) compared with that of the HFD group. An oral glucose tolerance test (OGTT) was performed at the end of the experiment (Figure 6D). The blood glucose level of the HFD group significantly was increased at 60 min after glucose loading and tended to increase at 120 min. The PW100 and PW300 groups had slightly reduced fasting blood glucose levels at 60 and 120 min after glucose loading. Compared to the HFD group, the blood glucose level was significantly reduced in the GC200 group at 120 min in response to the glucose load. Thus, PW300 and GC enhanced insulin sensitivity in obese mice by improving IPITT.

## 3. Discussion

Obesity is considered a serious public health issues in countries around the world, and its control is actively being discussed [17]. Our previous study revealed that an ethanol extract of PM had anti-obesity effects in vitro and in vivo [15]. The ethanol extract of PM stimulated expression of lipolysis, fatty acid (FA) oxidation, and brown fat-specific genes in epididymal WAT of obese mice [15]. Based on the previous study’s findings, this study aimed to explore whether the anti-obesity effects of PW were associated with its lipid molecular mechanism in WAT and/or BAT in HFD-induced obese mice.

The WAT anatomically consists two major adipose depots, subcutaneous WAT and visceral WAT [18]. An HFD results in an increase of whole-body fat and fat distribution, particularly fat deposition in visceral WAT [19,20]. Accumulation of visceral WAT is more closely associated with obesity-induced metabolic diseases than that of subcutaneous WAT [18]. A previous study showed that effective regulation of hypertrophy and hyperplasia in adipocytes expansion might be a potential therapeutic strategy for obesity [21]. In our study, PW and GC decreased the mean epididymal adipocyte size, indicating that PW and GC may directly affect a reduction in the size of adipocytes in WAT. In addition, food intake was reduced by the HFD with its high calorie density, but there was no significant change between the PW and GC administration. On the other hand, treatment with PW300 completely suppressed FER, total body weight gain, and WAT mass compared with those in the HFD group. FER is the body weight gain relative to the consumed food intake, and a low FER indicates that the body weight gain is small for the same amount of diet consumed. Consequently, the body weight loss effects of PW (300 mg/kg) are presumably due to the reductions of FER, total WAT mass, and epididymal WAT adipocyte size.

As an energy storage organ, WAT stores TG and releases FAs through lipogenesis and lipolysis, respectively [22]. Lipogenesis is a process that includes de novo FA synthesis and TG biosynthesis [22]. ChREBP is a metabolic regulator that modulates both lipid and glucose metabolism in adipose tissue [22,23,24], and ChREBP is mainly expressed in active sites of de novo lipogenesis such as liver, WAT, and BAT [25]. During the sequential esterification processes of FA, the DGAT2 enzyme catalyzes the last step of TG biosynthesis. In pharmacological studies, it has been established that inhibition of DGAT is a therapeutic approach to obesity and type 2 diabetes [26]. On the other hand, PPARα controls expression of genes related to FA oxidation and exhibits hypolipidemic effects [27,28]. Some studies have indicated that PPARα is expressed in the adipose tissue of humans and animals, indicating that adipose tissue might be a target organ of PPARα activators [27,29,30,31,32]. In this study, we observed that GC treatment (200 mg/kg) only decreased *DGAT2* expression, whereas PW treatment (300 mg/kg) significantly down-regulated *ChREBP* and *DGAT2* expression and slightly up-regulated *PPARα* expression in epididymal WAT, indicating partial mediation of reductions of adipocyte size and WAT mass. The WAT also secretes various adipokines for the control of whole-body energy homeostasis [18]. Obese WAT releases pro-inflammatory cytokines, including chemokines (e.g., MCP-1) [33]. A previous study showed that overexpression of MCP-1 in adipose tissue results in macrophage infiltration into adipose tissue, systemic insulin resistance, and fatty liver in obese mice [34]. The expressions of inflammatory genes (*NF-κB*, *TNF-α*, and *IL-6*) following PW treatment (100 and 300 mg/kg) tended to be lower than those in the HFD group, but the decreases were not statistically different. In addition, the PW100 and PW300 groups tended to decrease *MCP-1* gene expression compared with that of the HFD group. Hence, the reduction of *MCP-1* gene expression by PW treatment may have contributed to the prevention of obesity-induced inflammation.

Diet-induced thermogenesis occurs primarily in BAT and has a key role in regulating energy balance by burning off excess calories [35]. BAT contains UCP1, which uncouples energy substrate oxidation from mitochondrial ATP production, leading to the release of energy as heat [36]. BAT is activated in a UCP1-dependent manner and in the sympathetic nervous system (SNS) through ADRB3 [37,38]. Stimulation of SNS is driven by cold exposure, diet, stress, and inflammation, resulting in an increase in thermogenesis [37,38]. BAT uses FA and glucose as the main sources of energy for heat generation [4,39]. Activating ADRB3 enhances transcription of UCP1 and PGC-1α and accelerates the activity of lipolytic enzymes, leading to the release of FAs that enter the mitochondria for β-oxidation [40]. Briefly, activation of BAT secretes FAs through phosphorylation of adipose TG lipase (ATGL) and HSL, and the released FA is converted to heat [39]. Moreover, lipoprotein lipase (LPL) hydrolyzes TG-rich lipoproteins in the bloodstream for FA uptake and transport [41]. The released FA is taken up by CD36 and FA transfer proteins and is transferred to the cytoplasm by FABP4, also known as adipocyte protein 2 (aP2) [41]. Sustained β3-adrenergic receptor stimulation increases de novo lipogenesis, eventually stored as TG in lipid droplets [40]. A previous study reported that mice fed an HFD over 2–4 weeks had significantly increased CPT2, acyl-CoA thioesterase 2 (ACOT2), and UCP1 levels compared with mice fed a low-fat diet, but the increased protein levels were not maintained and returned to basal levels in HFD-fed mice for 20 weeks [5]. Unlike the above results, Li et al. [42] reported that mice fed an HFD for 22 weeks activated the catabolism of FA and the compensatory energy expenditure through the increase of CPT2 and UCP1 in BAT. In our results, the HFD group had elevated expressions of *ADRB3*, *PPARγ, HSL*, *CD36, FABP4*, *PGC-1α*, *PPARα*, and *CPT1B* genes in interscapular BAT. These results suggested that FA oxidation and lipolysis were up-regulated in response to fat overload. Interestingly, these gene expressions were restored by all of the tested concentrations of PW and GC. However, no significant changes were seen in the *UCP1* and *CPT2* gene expressions among the study groups. A recent study showed that an HFD stimulated FA β-oxidation by increasing *CPT1B* and *CPT2* expressions, whereas metformin (a drug for the treatment of type 2 diabetes) significantly down-regulated the *CPT1B* and *CPT2* expressions in BAT compared with HFD-fed mice, indicating that metformin might improve the energy metabolism of BAT and might reduce compensated energy expenditure [43]. As a result, the HFD-fed group exhibited blunt mitochondrial β-oxidation associated with a decrease in the ability to expend diet-derived energy as heat in response to a fat overload in BAT. The PW and GC treatment normalized diet-induced adaptive thermogenesis, and these changes were similar to a low dosage of PW (100 mg/kg) and GC (200 mg/kg). In the abnormal metabolic state, increased BAT gene expression via physiological compensation was recovered by PW and GC administration, potentially improving lipid metabolism.

An HFD can increase the TC serum level [19,44,45]. In this study, the PW300 group tended to have a reduced serum FFA level compared with that in the HFD group. Choi et al. [46] reported that, compared with a 0.5% PM ethanol extract intake, a 1% PM ethanol extract intake significantly reduced serum TG and TC levels in high-cholesterol diet-fed rats. Clinically, elevations of serum AST and ALT levels have been considered biomarkers for NAFLD [47]. In the present study, levels of AST and ALT, which are mostly produced in hepatocytes, were measured in serum to evaluate the degree of hepatocyte damage. HFD-fed mice had significantly increased serum ALT, which was attenuated by PW (100 and 300 mg/kg) and GC (200 mg/kg) treatments. A previous study demonstrated that oral administration of the aqueous extract of PM did not affect serum ALT and AST levels, even at a dosage of 40 g/kg for 3 days in rats [48]. Based on the results of Jung et al. [49], the no observed adverse effect level (NOAEL) of PM in rats for 2 weeks was estimated to be 1000 mg/kg. Our in vivo study also showed no symptoms of hepatotoxicity by PW treatment (100 and 300 mg/kg) for 12 weeks. PM contains various antioxidant compounds, including polyphenolic compounds and flavonoids [16]. The total phenol (85.43 ± 4.46 mg tannic acid equivalent (TE)/g) and flavonoid (1.57 ± 0.09 mg rutin equivalent (RE)/g) contents of the PW used in this study were higher than those (12.53 ± 0.02 mg/g, 0.10 ± 0.01 mg/g, respectively) reported by Kim et al. [50]; however, total phenol and flavonoid contents of the water extract were lower than those in PM extracted by other solvents (70% EtOH > 100% EtOH > distilled water). This is due to the higher polarity, compared to water, of other solvents used to extract phenolic compounds [51]. Noda et al. [52] showed that the PM hot water extract, which is the traditional and common PM preparation used for consumption, did not induce hepatotoxicity but did exhibit beneficial effects on liver function.

An HFD animal model could also be used to examine hyperinsulinemia and the development of insulin resistance [53,54,55]. Adipocytes may signal directly to beta cells to control insulin secretion and, therefore, could cause hyperinsulinemia regardless of blood glucose levels [56,57]. In this study, PW (300 mg/kg) and GC (200 mg/kg) treatments significantly reduced fasting serum insulin level without changing the fasting serum glucose level. Moreover, IPITT, which measures insulin sensitivity, was improved with PW (300 mg/kg) and GC (200 mg/kg) treatments. These data suggest that PW has the potential to protect against obesity-induced insulin resistance.

## 4. Materials and Methods

### 4.1. Preparation of the PW Extract

Extraction of PW was performed as previously described [58]. A previous study confirmed that PW contained 2,3,5,4′-tetrahydroxystilbene-2-β-glucoside, emodin, and physcion [58]. Briefly, the PM was obtained from Dong-Bu Herbal Marker (Suncheon, Korea). PM (500 g) was extracted repeatedly with water (3 × 5 L) and exhaustive maceration at 85 °C for 3 h. The supernatant was filtered and transferred into pre-weighed containers. The extract was concentrated by a rotary evaporator and then freeze-dried to obtain the crude extract (170 g).

### 4.2. Animal Experiments

Male C57BL/6N mice (4-week-old) were purchased from Orient Bio, Ltd. (Seoul, Korea). All mice were housed in individual cages under controlled temperature (22 ± 2 °C), humidity (50 ± 5%), and 12 h light–dark cycle conditions. After a one week adaptation period, the mice were randomly divided into five groups and received a normal diet (NCD, *n* = 9, 10 kcal% fat, cat #D10012G, Research Diets, Inc., New Brunswick, NJ, USA), HFD (*n* = 11, 45 kcal% fat, cat #D12451, Research Diets, Inc.), HFD with oral administration of PW at 100 mg/kg body weight (PW100, *n* = 10), HFD with PW at 300 mg/kg body weight (PW300, *n* = 10), or HFD with garcinia cambogia (GC) at 200 mg/kg body weight (GC200, *n* = 8, Shinwon, Anyang, Korea) for 12 weeks. GC is used a dietary supplement to reduce body fat and has major active ingredients, such as hydroxycitric acid [59]. Mice were provided experimental diet and tap water ad libitum. Body weight and food intake were measured weekly and every 2–3 days, respectively, during the feeding period. FER was calculated using the following formula: body weight gain/food intake per day.

At the end of the 12-week treated period, mice were fasted for 12 h, then anesthetized with ether and sacrificed. Blood samples were subsequently taken from the inferior vena cava for serum biomarker analysis. The epididymal, retroperitoneal, perirenal, mesenteric, subcutaneous, and interscapular WAT, and the interscapular BAT were excised immediately, rinsed with physiological saline, and weighed. All samples were stored at −80 °C until analysis. The experimental procedures were approved by the Sunchon National University Institutional Animal Care and Use Committee (SCNU IACUC-2017-04).

### 4.3. Intraperitoneal Insulin Tolerance Test and Oral Glucose Tolerance Test

IPITT and OGTT analyses were performed at 11 weeks and 12 weeks, respectively. For the IPITT, the mice were injected intraperitoneally with insulin (1 unit/kg body weight) after 6 h of fasting. The fasting blood glucose levels were determined by using samples collected from the tail vein at 0, 30, 60, and 90 min after the insulin injection. For the OGTT, the mice were fasted for 6 h and then given glucose orally (1 g/kg body weight), and the fasting blood glucose levels were measured after 0, 30, 60, and 120 min.

### 4.4. Serum Markers Levels and Lipid Contents

Serum ALT and AST activities were measured using an automated chemistry analyzer (Fuji Dri-Chem 3500i, Fujifilm, Tokyo, Japan). Serum fasting glucose concentration was measured using a glucometer (G-doctor, AllMedicus, Co., Ltd., Anyang, Korea). Serum insulin level was determined by an ELISA kit (Morinaga Institute of Biological Science, Inc., Yokohama, Japan). The HOMA-IR was calculated as fasting serum insulin (µIU/mL) × fasting serum glucose (mmol/L)/22.5. The serum TC, HDL-C, TG (Asan Pharmaceutical Co., Ltd., Seoul, Korea), and FFA (Shinyang Diagnostics, Seoul, Korea) contents were measured using commercial kits. The HTR was calculated as HDL-C/TC × 100.

### 4.5. Histological Analysis

Epididymal WAT was fixed with 10% neutral-buffered formalin. The fixed tissues were paraffin-embedded, and 3–5 µm sections were prepared and stained with hematoxylin and eosin (H&E). The stained area was then viewed using an optical microscope at 200× magnification.

### 4.6. RNA Isolation and Quantitative Real-Time PCR (qRT-PCR) Analysis

Total RNA was isolated from WAT and BAT using TRIzol reagent (Invitrogen, Carlsbad, CA, USA). RNA purity and integrity were evaluated using a Nanodrop 2000 spectrophotometer (Thermo Fisher Scientific, Waltham, MA, USA). The RNA was reverse transcribed into cDNA using a ReverTra Ace qPCR RT master mix (Toyobo, Osaka, Japan). qRT-PCR was performed using a SYBR green PCR kit (Qiagen, Hilden, Germany) and a CFX96TM real-time detection system (Bio-Rad Laboratories, Inc., Hercules, CA, USA). The primer sequences are shown in Table 3. The relative mRNA levels were measured by applying the 2^−△△Ct^ method [60] and were normalized to the ribosomal protein lateral stalk subunit P0 (*RPLP0*) expression level in the same samples.

### 4.7. Statistical Analysis

Values are presented as means ± standard error (SE) values. Statistical significance among groups were determined by one-way analysis of variance (ANOVA) and Tukey’s HSD test using the Statistical Package for the Social Sciences (SPSS version 26, SPSS Inc., Chicago, IL, USA). A *p*-values less than 0.05 were considered as statistically significant.

## 5. Conclusions

In conclusion, PW (300 mg/kg) significantly ameliorated body weight gain, body fat, and insulin resistance in HFD-fed obese mice. These beneficial effects were accompanied by changes in lipid metabolism-related gene expressions in WAT and BAT. The PW treatment inhibited lipogenesis in WAT. Furthermore, PW normalized gene expressions related to HFD-induced adaptive thermogenesis in BAT. These results reveal that PW is a useful agent against obesity and obesity-associated metabolic diseases, and its effects are similar to those of GC.

## Figures and Tables

**Table 1:** Effects of *Polygonum multiflorum* Thunb. hot water extract on body weight in HFD diet-induced obese mice.

	NCD	HFD	PW100	PW300	GC200
Body weight (g)					
0 weeks	20.26 ± 0.23	20.55 ± 0.35	20.18 ± 0.24	20.34 ± 0.31	20.39 ± 0.31
1 weeks	21.61 ± 0.36	22.50 ± 0.36	21.19 ± 0.54	20.96 ± 0.42	22.35 ± 0.36
2 weeks	22.46 ± 0.41	23.64 ± 0.35	22.30 ± 0.45	22.28 ± 0.61	24.03 ± 0.27
3 weeks	23.20 ± 0.45	24.67 ± 0.32	23.83 ± 0.54	23.52 ± 0.54	24.65 ± 0.36
4 weeks	24.30 ± 0.56	26.18 ± 0.32	24.75 ± 0.50	24.64 ± 0.60	26.28 ± 0.48
5 weeks	25.13 ± 0.70	27.06 ± 0.42	25.67 ± 0.48	24.90 ± 0.65	26.69 ± 0.48
6 weeks	26.53 ± 0.75 ^ab^	28.84 ± 0.44 ^b^	26.97 ± 0.53 ^ab^	26.33 ± 0.56 ^a^	28.14 ± 0.58 ^ab^
7 weeks	27.50 ± 0.65 ^a^	30.35 ± 0.41 ^c^	28.20 ± 0.55 ^ab^	27.92 ± 0.50 ^a^	30.06 ± 0.38 ^bc^
8 weeks	27.70 ± 0.91 ^a^	31.35 ± 0.49 ^c^	28.98 ± 0.61 ^abc^	28.10 ± 0.55 ^ab^	30.23 ± 0.42 ^bc^
9 weeks	28.65 ± 0.97 ^a^	32.41 ± 0.51 ^b^	30.14 ± 0.76 ^ab^	28.75 ± 0.65 ^a^	30.95 ± 0.50 ^ab^
10 weeks	30.22 ± 0.91 ^a^	34.45 ± 0.54 ^b^	31.68 ± 0.78 ^ab^	30.62 ± 0.69 ^a^	32.10 ± 0.59 ^ab^
11 weeks	31.34 ± 0.88 ^a^	35.71 ± 0.50 ^b^	33.39 ± 0.76 ^ab^	32.04 ± 0.85 ^a^	33.09 ± 0.63 ^ab^
12 weeks	31.85 ± 0.89 ^a^	36.54 ± 0.51 ^b^	34.42 ± 0.83 ^ab^	33.16 ± 0.77 ^a^	33.73 ± 0.57 ^ab^
Body weight gain (g)	11.61 ± 0.75 ^a^	16.00 ± 0.61 ^b^	14.23 ± 0.63 ^ab^	12.82 ± 0.71 ^a^	13.33 ± 0.40 ^a^

Data are expressed as means ± SE. Values with different superscript letters are significantly different at *p* < 0.05 as determined by one-way ANOVA followed by Tukey’s HSD test.

**Table 2:** Effects of *Polygonum multiflorum* Thunb. hot water extract on food intake, food efficiency ratio, serum marker levels, and lipid contents in HFD diet-induced obese mice.

	NCD	HFD	PW100	PW300	GC200
Food intake (g/day)	3.24 ± 0.05 ^b^	2.83 ± 0.03 ^a^	2.73 ± 0.02 ^a^	2.72 ± 0.03 ^a^	2.75 ± 0.03 ^a^
FER	0.0423 ± 0.0022 ^a^	0.0671 ± 0.0021 ^c^	0.0619 ± 0.0025 ^bc^	0.0558 ± 0.0025 ^b^	0.0576 ± 0.0013 ^b^
Serum marker levels					
AST (U/L)	54.62 ± 2.92	62.60 ± 3.06	62.00 ± 3.86	52.40 ± 2.07	54.87 ± 2.96
ALT (U/L)	23.12 ± 2.01 ^a^	34.40 ± 2.70 ^b^	32.10 ± 3.55 ^ab^	24.50 ± 2.12 ^ab^	28.00 ± 2.34 ^ab^
Glucose (mmol/L)	11.76 ± 1.37	12.95 ± 0.73	10.64 ± 0.97	11.07 ± 1.01	13.54 ± 0.68
Serum lipid contents					
Triglyceride (mmol/L)	0.80 ± 0.05	0.93 ± 0.05	0.94 ± 0.03	0.93 ± 0.05	0.83 ± 0.02
Free fatty acid (mmol/L)	0.84 ± 0.05 ^b^	0.79 ± 0.07 ^b^	0.76 ± 0.03 ^b^	0.65 ± 0.05 ^ab^	0.43 ± 0.05 ^a^
Total cholesterol (mmol/L)	3.96 ± 0.17	4.60 ± 0.16	4.27 ± 0.16	4.13 ± 0.09	4.40 ± 0.14
HDL-cholesterol (mmol/L)	2.56 ± 0.05 ^a^	3.01 ± 0.10 ^b^	2.97 ± 0.09 ^ab^	2.90 ± 0.05 ^ab^	3.21 ± 0.19 ^b^
HTR (%)	65.38 ± 2.52	66.92 ± 2.02	69.79 ± 1.52	69.30 ± 1.11	74.62 ± 4.61

Data are expressed as means ± SE. Values with different superscript letters are significantly different at *p* < 0.05 as determined by one-way ANOVA followed by Tukey’s HSD test. Food efficiency ratio (FER) = body weight gain (g/day)/food intake (g/day). AST, aspartate aminotransferase; ALT, alanine aminotransferase. HTR = HDL-cholesterol/total cholesterol × 100.

**Table 3:** Primer sequences for qRT-PCR.

Gene	Full Name	Sequences of Forward and Reverse Primer (5′-3′)
*PGC-1α*	Peroxisome proliferative activated receptor, gamma, coactivator 1 alpha	GTCATGTGACTGGGGACTGTAG/TCCACTCTGACACACAGCAC
*PPARα*	Peroxisome proliferator-activated receptor alpha	GCTGGAGGGTTCGTGGAGTC/CGGTGAGATACGCCCAAATGC
*UCP1*	Uncoupling protein1 (mitochondrial, proton carrier)	CCTGCCTCTCTCGGAAACAA/TCTGGGCTTGCATTCTGACC
*CPT1B*	Carnitine palmitoyltransferase 1b	TGGCTACGGGGTCTCTTACA/AAGTTCGGCGATGTCCAACA
*CPT2*	Carnitine palmitoyltransferase 2	GCCTGCTGTTGCGTGACTG/TGGTGGGTACGATGCTGTGC
*HSL*	Lipase, hormone sensitive	GTGAATGAGATGGCGAGGGTC/TGAGGAGTCGCGTTAGAGTC
*PPARγ*	Peroxisome proliferator-activated receptor gamma	TCGCTGATGCACTGCCTATG/GAGAGGTCCACAGAGCTGAT
*C/EBPα*	CCAAT/enhancer binding protein (C/EBP), alpha	GCGCAAGAGCCGAGATAAA/GGTGAGGACACAGACTCAAATC
*SREBP1c*	Sterol regulatory element binding transcription factor 1	AACCTCATCCGCCACCTG/TGGTAGACAACAGCCGCATC
*ADRB3*	Adrenergic receptor, beta 3	CAGGCTCTGTGTCTCTGGTTAG/GTGAGGAGACAGGGATGAAACC
*ChREBP*	Carbohydrate-responsive element-binding protein	GAAGGAATGGGTCCAGACATAC/TCACACTGGTCACTCCTACA
*CD36*	CD36 antigen	GCTGTCAGGCGTCAGGATAA/TGGCTTCAGGGAGACTGTTG
*FABP4*	Fatty acid binding protein 4, adipocyte	TTTGGTCACCATCCGGTCAG/CCCGCCATCTAGGGTTATGA
*FAS*	Fatty acid synthase	TTGGAGCTAAGGCATGGTGG/GCAGTTGTCCTCTGGATGCT
*SCD1*	Stearoyl-coenzyme A desaturase 1	TTCTTCATCGACTGCATGGC/ACTCAGAAGCCCAAAGCTCAG
*DGAT2*	Diacylglycerol O-acyltransferase 2	CTGGCTGATAGCTGTGCTCTACTTC/TGCGATCTCCTGCCACCTTTC
*NF-κB*	Nuclear factor of kappa light polypeptide gene enhancer in B cells	GAAGTGAGAGAGTGAGCGAGAGAG/CGGGTGGCGAAACCTCCTC
*TNF-α*	Tumor necrosis factor	AAAGACACCATGAGCACAGAAAGC/GCCACAAGCAGGAATGAGAAGAG
*IL-6*	Interleukin 6	AGTCCTTCCTACCCCAATTTCC/TGGTCTTGGTCCTTAGCCAC
*MCP1*	Chemokine (C–C motif) ligand 2	GAAGGAATGGGTCCAGACATAC/TCACACTGGTCACTCCTACA
*RPLP0*	Ribosomal protein, large, P0	GCAGGTGTTTGACAACGGCAG/GATGATGGAGTGTGGCACCGA

## Data Availability

The data presented in this study are available on request from the corresponding author.
